# The Role of Pulmonary Function Test in Perioperative Management of Patients with Cystic Fibrosis

**Published:** 2022-06-06

**Authors:** Mariel Manzor, Gabor Asztalos, Koichi Yuki

**Affiliations:** Department of Anesthesiology, Critical Care and Pain Medicine, Cardiac Anesthesia Division, Boston Children’s Hospital, USA

## Abstract

Cystic fibrosis (CF) is one of chronic illness affecting many different organs. Although the outcome of CF patients undergoing procedures was not favorable more than half a century ago, it showed a continuous improvement based on the previous reports. However, recent outcome report of CF patients’ procedural outcomes is limited. We analyzed CF patients who underwent procedures from 2010 to 2020 in our institution. The mortality of 1,903 procedures performed in 430 CF patients was 0.74%, seen in high-risk procedures such as lung transplantation. We also analyzed the perioperative profiles of CF patients who underwent functional endoscopic sinus surgery (FESS). We identified the preoperative pulmonary function testing result to be predictive of postoperative hospital stay.

## Background

Cystic Fibrosis (CF) is a multisystem disorder resulting from pathogenic mutations in the CF transmembrane conductance regulator (CFTR) gene. The CFTR protein transports chloride and bicarbonate across the apical surface of secretory epithelia in various tissues and organs including lung and pancreas [1,2]. CF-causing CFTR mutations lead either to diminished or defective CFTR protein, resulting in decreased chloride transport. The impairment of chloride transport causes thick and viscous mucus to accumulate, causing a variety of complications including chronic lung infections, pancreatic and liver dysfunction, and reduced fertility [3].

Patients with CF not only receive medical treatment but also undergo a multitude of surgeries for CF-related or non-related medical issues [4]. Earlier studies demonstrated the drastic improvement of surgical outcome in CF patients; Salanitre, et al. reported the perioperative mortality of 27% in 135 surgeries for CF patients from 1945 to 1962 [5]. In the series from 1959 to 1970 by Doershuk, et al., there was a mortality rate of 4% in 144 surgical procedures [6]. Olsen, et al. reported a significantly reduced mortality rate of 0.5% in 578 surgeries from 1970 to 1985 [7].

The improvement of CF care has steadily increased the life-expectancy. Over 50% of CF patients reach adulthood, and those in high income countries live longer than 40 years [8]. As surgical, medical treatment, and anesthetic management have continued to show an improvement, it is not infrequent to see more complex procedures performed. Because recent literature analyzing perioperative outcomes in CF patients is limited, our primary objective was to examine the type of procedures and the outcomes of CF patients. In addition, we determined the risk factors contributing to longer hospital stays following one of common surgical procedures functional endoscopic sinus surgery (FESS) in CF patients.

## Methods

### Patients and data collection

Following the institutional review board (IRB) approval, the retrospective chart review was performed. Informed consent was exempted. We identified procedures performed for patients with CF at Boston Children’s Hospital between January 2010 and December 2020. CF was diagnosed with a sweat test and/or a blood-based genetic test. To examine the type of procedures that CF patients underwent from 2010 to 2020, we used the electronic medical record to obtain patient demographics, and type of procedures. We included all the CF patients who underwent procedures during the study period. To further study the recovery characteristics of CF patients undergoing one of the common procedures FESS, we also extracted their American Society of Anesthesia (ASA) physical status classification, postoperative length of hospital stays, and pulmonary function tests (PFTs) within 6 months preoperatively and postoperatively. In addition, we sought for the presence of CF related complications as well as the type of CFTR mutations. For genetic makeup, homozygous corresponds to patients having two F508del mutations, heterozygous corresponds to patients having one F508del mutation [9], and unknown corresponds to patients whose genetic mutation was unknown from the medical record. For the study of FESS, we excluded patients who did not have perioperative PFTs. We also did not include patients who already received lung transplantation at the time of FESS.

### Statistical analysis

We report categorical variables as number and percentage, and continuous variables as mean and standard deviation for normally distributed variables or median and interquartile range for variables with skewed distribution. Normality was examined using Shapiro-Wilk normality test. We compared the proportions of categorical variables with *λ*^2^ test and assessed the distribution of continuous variables with independence sample *t* test (student *t* test) or Mann-Whitney test for potential risk factors for prolonged hospital length of stay postoperatively. The results were also expressed as odds ratio [1] and 95% confidence interval (CI). Cutoff values were determined using Youden-J statistics. Analysis was performed using STATA (College Station, TX).

## Results

### Characteristics of CF patients undergoing procedures and the type of procedures

**Figure 1:**
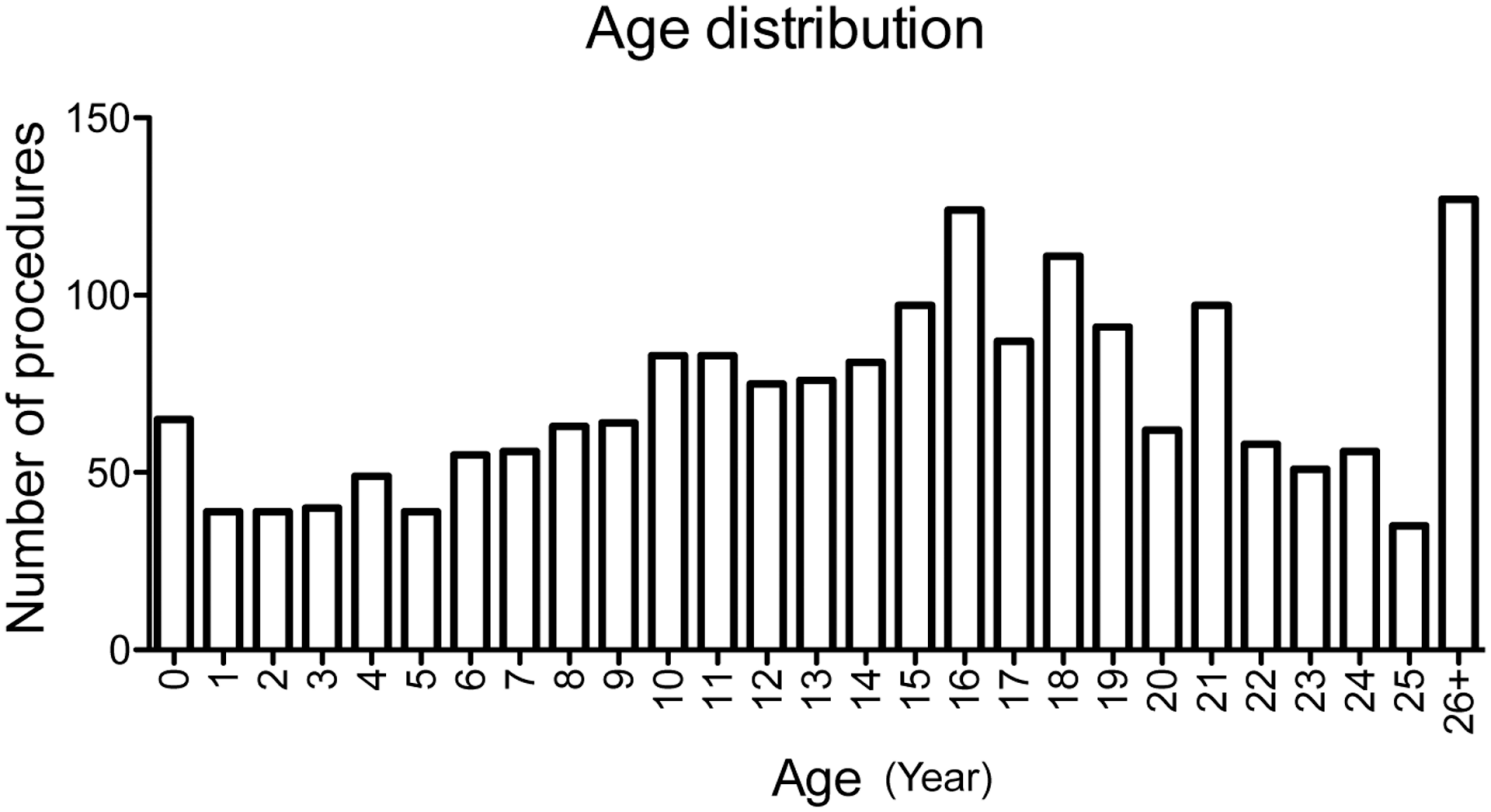
Age distribution and number of procedures. The relationship between age (year) of cystic fibrosis (CF) patients and the number of procedures from 2010 to 2020 was shown.

We identified 1,903 procedures performed in 430 CF patients during the study period (Table 1). Median age was 15-years-old, with roughly equal number of males and females. Age distribution was shown in Figure 1. Highest number of procedures was peaked at age of 16 years in our cohort. 5 patients died during the same admission following procedures. These 5 patients had total of 14 procedures during the admission, indicating 0.74% of procedural mortality rate.

**Figure 2:**
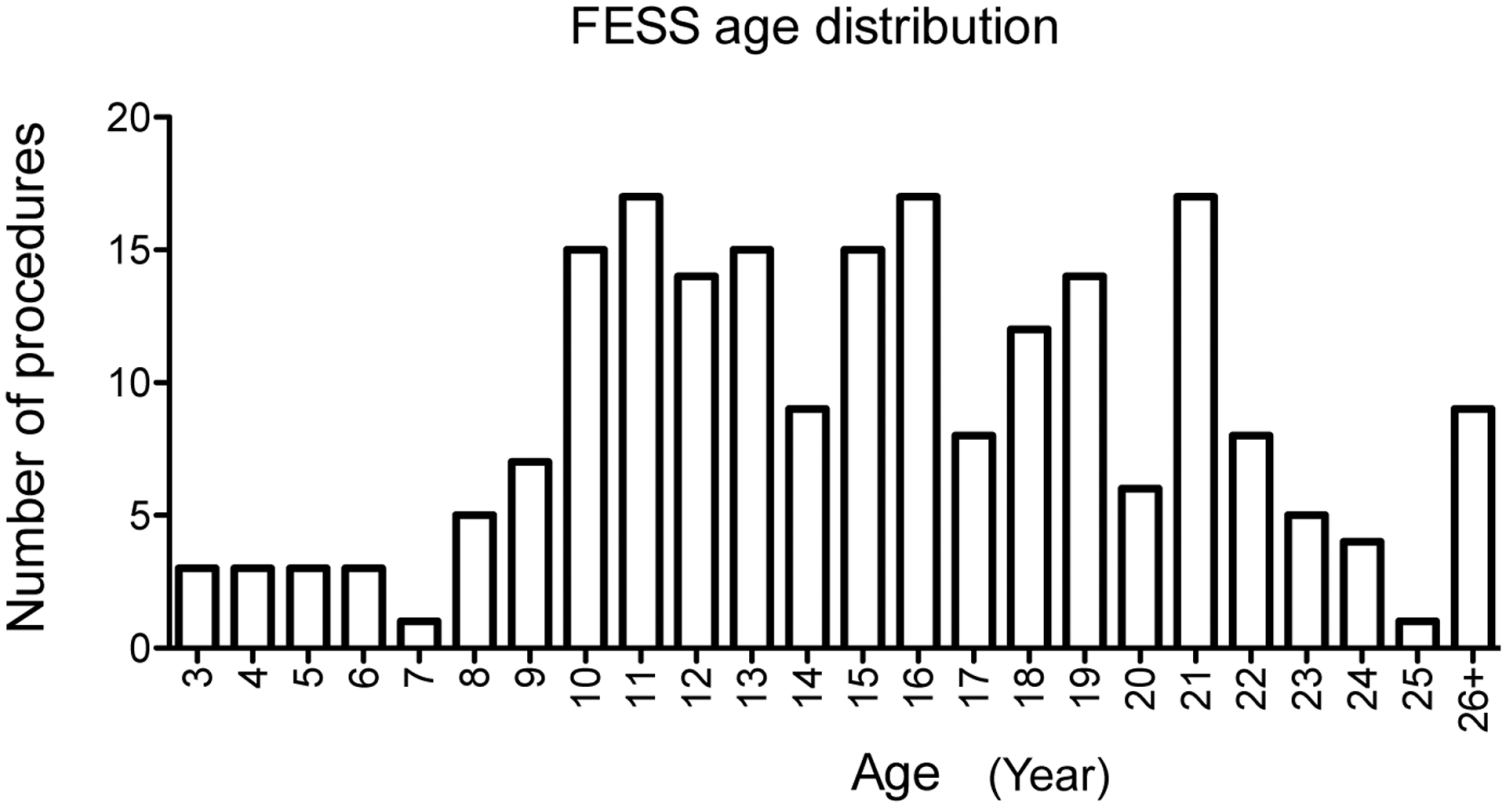
Age distribution and number of FESS procedures. The relationship between age (year) of CF patients and the number of FESS procedures from 2010 to 2020 was shown. FESS: Functional endoscopic sinus surgery.

Table 2 showed the summary of procedures. Peripherally inserted central catheter (PICC) line placement was the most frequent procedure, followed by flexible bronchoscopy and bronchioalveolar lavage and FESS. Table 3 showed common procedures per different age group. While PICC line was the most common procedure among all age groups, FESS was more commonly performed after 10-years-old (Figure 2).

### The recovery profiles of CF patients undergoing FESS

**Figure 3:**
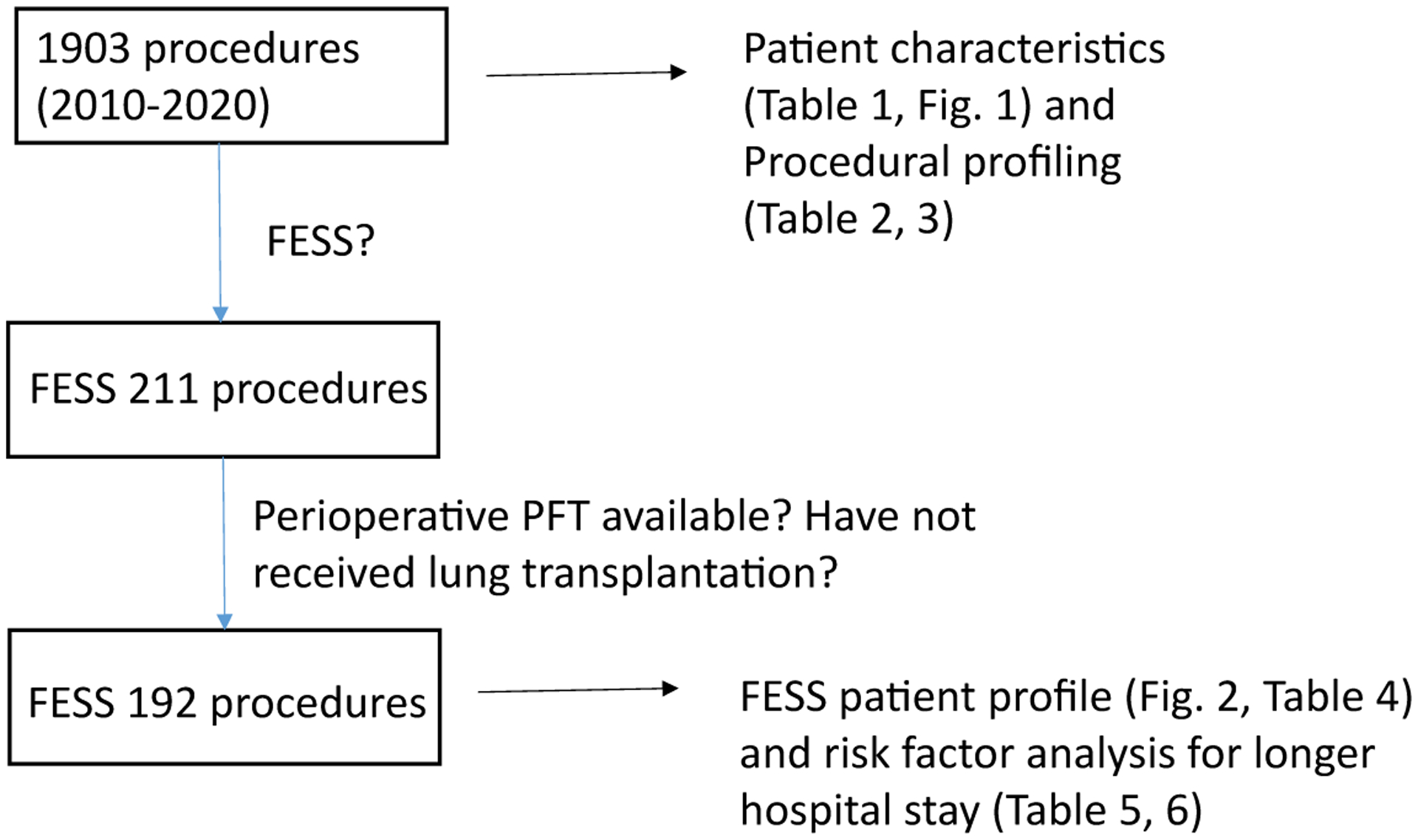
The flow diagram of identifying patients who underwent FESS procedures for analysis FESS: Functional endoscopic sinus surgery

Because PICC line is a less invasive procedure compared to others, we chose to study the perioperative characteristics of CF patients undergoing FESS (Figure 3). We identified 119 patients who underwent total of 211 FESS procedures (Table 4). Median age was 15-years-old. No mortality was observed in the cohort. 192 of patients undergoing FESS had PFTs within 6 months before and after the surgery (Figure 3). Usually patients who undergo FESS procedures are discharged home on the same day or next day. We determined the factors associated with hospital stays ≥ 2 days following the procedure. We identified 62 patients who stayed ≥ 2 days (Group B) and 130 patients who were discharged home by the following day after surgery (Group A). Patients in Group B were slightly older by average (Table 5). BMI was lower in Group B. The PFT values in Group B were worse compared to the ones in Group A. On the other hand, gender, weight, ASA status, the complexity of FESS, the type of genetic mutations, and the number and type of CF related non-respiratory complications were not associated with longer hospital stay. We further determined the cutoff value of the parameters associated with longer hospital stay using Youden-J statistics. The cutoffs which determine whether a patient is likely to spend two or more nights in the hospital following FESS procedure were as follows: ≥ 18-years-old, Pre-%FVC ≤ 87, Pre-%FEV_1_ ≤ 81, Pre-%FEV_1_/FVC ≤ 83, Pre-%FEF_25–75_ ≤ 60, Post-%FVC ≤ 92, Post-%FEV_1_ ≤ 81, Post-%FEV_1_/FVC ≤ 80, and Post-%FEF_25–75_ ≤ 58 (Table 6). Given that each PFT parameter was shown helpful to predict hospital stays ≥ 2 days, we also examined the correlation between the duration of hospital stay and each PFT parameter using linear regression analysis. As predicted, there was a trend that each PFT parameter value was lower in patients with longer hospital stay, although r^2^ value was relatively low (Supplemental Figure 1).

## Discussion

Our single institutional data analysis showed the mortality rate of CF patients following procedures was 0.74%, similar to what Olsen, et al. reported using the data from 1970–1985. In the study by Olsen, et al. the majority of procedures were nasal polypectomy and vascular access procedures in patients with the median age of 16 years, which is quite similar to the characteristics of our cohort. However, our cohort also included more invasive procedures such as liver and lung transplantations. In the Olsen cohort, three patient died (3/578 cases, 0.52%); One underwent lobectomy and two underwent surgeries for meconium ileus. In our cohort, two patients died following lung transplantation, two patients following tracheostomy and one after flexible bronchoscopy. Patients who underwent lung transplantation had multiple procedures following lung transplantations. Because some of our procedural profiles were not exactly the same with ones in Olsen study, it is difficult to simply compare two results from the mortality standpoint. However, it is safe to say that the outcome of CF patients following most of procedures is overall good from the mortality perspective.

Acknowledging their overall favorable outcomes, it is important to understand their perioperative profiles. For this purpose, we examined the perioperative course of patients undergoing FESS, realizing that some of CF patients required hospital stays more than one night. PFTs are not required as a routine preoperative testing in the majority of surgical procedures. Our analysis showed that PFT could be a useful predictive test for the longer duration of postoperative stay after surgical procedures in CF patients. We presented the cutoff values that likely help to determine patients who require hospital stay > or = 2 nights following the procedures. Age was also a risk factor, which likely indicates the progressive nature of CF disease in the lungs. Interestingly, the presence and the type of non-respiratory CF related complications did not show any association with longer postoperative hospital stay.

Although our study describes current perioperative status of CF patients, it has some limitations. First, this is retrospective in nature. Second, our study only represents our institutional experience and further investigation across other institutions is necessary. Third, we examined the use of PFTs only in FESS procedures. Analyzing other procedures will be important to see if our observation is relevant to many procedures.

In conclusion, the outcome of CF patients is overall favorable. In our institutional data, PFT may be a useful testing to be included in CF patients to predict their postoperative length of stay.

## Supplementary Material

1

## Figures and Tables

**Table 1:** The characteristics of cystic fibrosis patients.

Total patients	430 patients
Age	15.0 (9.0, 20.0)
Gender	Male 223, Female 207
Race	White 357 (83.0%)
Total procedures	1903 cases
Average procedures/patient	4.4 cases

**Table 2:** Procedural profiles of cystic fibrosis patients.

Type of procedures	Number of cases
PICC line placement	602 (31.63%)
Flexible bronchoscopy/bronchioalveolar lavage	217 (11.40%)
FESS (limited, complex)	211 (11.09%)
Esophagogastroduodenoscopy	133 (6.99%)
Port-A-cath placement	81 (4.26%)
Gastrostomy	43 (2.26%)
Colonoscopy	42 (2.21%)
Cystoscopy	34 (1.79%)
Laparotomy	30 (1.58%)
Direct laryngoscopy and/or bronchoscopy	29 (1.52%)
Lung transplant	20 (1.05%)
Hernia repair	17 (0.89%)
Gastrocutaneous fistula closure	11 (0.58%)
Liver transplant recipient	10 (0.53%)
Arthroscopy (ankle, knee, elbow, or shoulder)	10 (0.53%)
Laparoscopic Appendectomy	8 (0.42%)
Others	405 (21.28%)
Total	1903

PICC: Peripherally Inserted Central Catheter; FESS: Functional Endoscopic Sinus Surgery

**Table 3:** Procedural profiles per age group.

Type of procedures	Number of cases
**0–5 years**	**271**
PICC line placement	40 (14.76%)
Flexible bronchoscopy/bronchioalveolar lavage	23 (8.49%)
Esophagogastroduodenoscopy	21 (7.75%)
Laparotomy	19 (7.01%)
Gastrostomy	17 (6.27%)
Direct laryngoscopy and/or bronchoscopy	15 (5.54%)
FESS	9 (3.32%)
Tonsillectomy/Tonsillotomy	8 (2.95%)
Colonoscopy	7 (2.58%)
Hernia repair	7 (2.58%)
6–10 years	321
PICC placement	73 (22.4%)
Flexible bronchoscopy/bronchioalveolar lavage	47 (14.64%)
Esophagogastroduodenoscopy	29 (9.03%)
FESS	31 (9.66%)
Port-A-cath placement	11 (3.43%)
Lung transplant	7 (2.18%)
11–15 years	412
PICC placement	134 (32.52%)
FESS	70 (16.99%)
Flexible bronchoscopy/bronchioalveolar lavage	52 (12.62%)
Esophagogastroduodenoscopy	36 (8.74%)
Colonoscopy	11 (2.67%)
Gastrostomy	9 (2.18%)
Port-A-cath placement	8 (1.94%)
16–20 years	475
PICC placement	157 (33.05%)
Flexible bronchoscopy/bronchioalveolar lavage	68 (14.32%)
FESS	57 (12.00%)
Esophagogastroduodenoscopy	19 (4.00%)
Odontectomy	15 (3.16%)
Port-A-cath placement	13 (2.74%)
Cystoscopy	10 (2.11%)
Arthroscopy (ankle, knee, elbow, or shoulder)	10 (2.11%)
21–25 years PICC placement	297 126 (42.42%)
FESS	35 (11.78%)
Flexible bronchoscopy/bronchioalveolar lavage	20 (6.73%)
Esophagogastroduodenoscopy	20 (5.73%)
Cystoscopy	14 (4.71%)
26 years-	127
PICC placement	69 (54.33%)
FESS	9 (7.09%)
Cystoscopy	8 (6.30%)
Flexible bronchoscopy/bronchioalveolar lavage	8 (6.30%)

PICC: Peripherally Inserted Central Catheter; FESS: Functional Endoscopic Sinus Surgery

**Table 4:** The characteristics of cystic fibrosis patients undergoing FESS procedures.

Total patients	119 patients
Age	15.0 (11.0, 19.0)
Gender	Male 59, Female 60
Race	White 107 (89.9%)
Total procedures	211 cases
Average procedures/patient	1.8 cases

FESS: Functional endoscopic sinus surgery

**Table 5:** Risk factor analysis of longer hospital stay following FESS procedures.

	Hospital stay < 2 days n = 130	>= 2 days n = 62	O.R.	95% CI	p value
Age	15.01+/− 5.15	17.32+/− 6.04	1.08	1.03–1.14	0.008
Gender	male 64 (49.23%) female 66 (50.77%)	male 28 (45.16%) female 34 (54.84%)	0.85	0.46–1.56	0.598
Weight	49.57+/− 15.01	50.33 +/− 14.17	1.004	0.98–1.02	0.737
BMI	20.00 (18.18, 22.50)	19.00 (16.50, 21.00)	0.887	0.80–0.99	0.026
FESS type	limited 70 (53.85%) complex 60 (46.15%)	limited 28 (45.16%) complex 34 (54.84%)	0.71	0.38–1.30	0.261
ASA	Class I 0 (0%)	Class I (0%)	1.87	0.96–3.67	0.067
	Class II 47 (36.15%)	Class II 15 (24.19%)			
	Class III 83 (63.85%)	Class III 46 (74.19%)			
	Class IV (0%)	Class IV (1.61%)			
Pre-%FVC	101.0 (91.0, 110.0)	84.0 (63.75, 101.5)	0.96	0.94–0.97	<0.001
Pre-%FEV1	94.0 (81.75, 109.0)	73.50 (45.75, 93.8)	0.97	0.95–0.98	< 0.001
Pre-%FEV1/FVC	88.0 (83.0, 95.0)	84.0 (72.0, 92.3)	0.95	0.93–0.98	< 0.001
Pre-%FEF25–75	82.0 (61.0, 105.0)	51.5 (21.0, 81.0)	0.98	0.96–0.98	< 0.001
Post-%FVC	98.5 (87.8, 111.0)	84.0 (60.8, 98.5)	0.96	0.94–0.98	< 0.001
Post-%FEV1	94.0 (80.0, 106.0)	75.0 (42.0, 93.3)	0.97	0.96–0.98	< 0.001
Post-%FEV1/FVC	88.0 (82.0, 95.0)	85.0 (69.8, 91.0)	0.96	0.94–0.99	0.002
Post-%FEF25–75	83.0 (55.8, 104.0)	55.0 (22.8, 84.0)	0.98	0.97–0.99	< 0.001
Genetic	Homozygous 37 (28.46%)	Homozygous 17 (27.42%)	1.02	0.47–2.20	0.969
	Heterozygous 45 (34.62%)	Heterozygous 21 (33.87%)			
	Unknown 48 (36.92%)	Unknown 24 (38.71%)			
CF non-respiratory complications	1 (1, 2)	2 (1, 2)	1.11	0.77–1.58	0.58
CF non-respiratory complication number	0–23 (17.69%)	0–11 (17.74%)			
	1–50 (38.46%)	1–19 (30.63%)			
	2–48 (36.92%)	2–28 (45.16%)			
	3–9 (6.92%)	3–4 (4.84%)			
Chronic sinusitis	83 (63.36%)	40 (64.52%)	1.03	0.55–1.94	0.928
Pancreatic insufficiency	78 (59.54%)	41 (66.13%)	1.3	0.69–2.44	0.414
Liver disease	12 (9.16%)	6 (9.68%)	1.05	0.38–2.95	0.921

FESS: Functional Endoscopic Sinus Surgery; ASA: American Society of Anesthesiologists; FVC: Forced Vital Capacity; FEV1: Forced Expiratory Volume in the First Second; FEF25–75: Forced Expiratory Flow At 25–75% Of The Forced Vital Capacity; CF: Cystic Fibrosis

**Table 6:** Cutoff values of PFTs in cystic fibrosis patients who had hospital stays > or = 2 days after FESS procedures.

	Cutoff value	AUC
Age	> = 18-year-old	0.61
Pre-%FVC	< = 87	0.71
Pre-%FEV_1_	< = 81	0.73
Pre-%FEV_1_/FVC	< = 83	0.63
Pre-%FEF_25–75_	< = 60	0.73
Post-%FVC	< = 92	0.71
Post-%FEV_1_	< = 81	0.71
Post-%FEV_1_/FVC	< = 80	0.63
Post-%FEF_25–75_	< = 58	0.69

PFT: Pulmonary Function Test; FESS: Functional Endoscopic Sinus Surgery; AUC: Area under the Curve; FVC: Forced Vital Capacity; FEV1: Forced Expiratory Volume in the First Second; FEF25–75: Forced Expiratory Flow at 25–75% of the Forced Vital Capacity; CF: Cystic Fibrosis
