# Supplementary material for: The Role of Pulmonary Function Test in Perioperative Management of Patients with Cystic Fibrosis
Source: Transl Perioper Pain Med. Author manuscript; Available in PMC 2022 Jul 21. (PMC9302475)
Supplement: 1 [file NIHMS1823235-supplement-1.pdf]

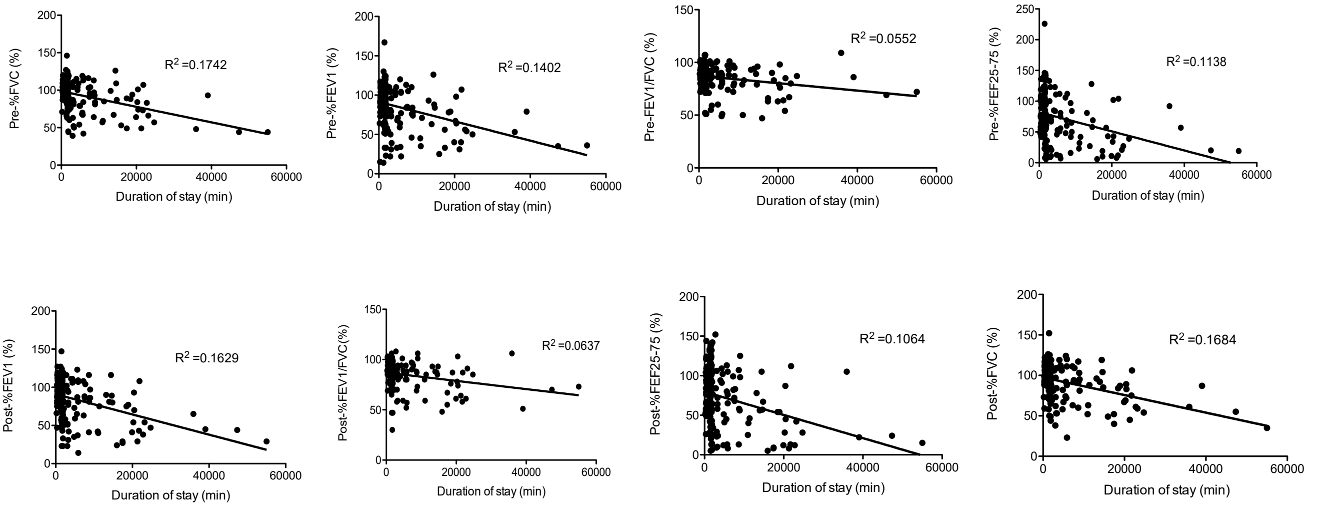

Supplemental Figure 1: The relationship between the duration of postoperative hospital stayand PFTs in patients who underwent FESS. X-axis and y-axis showed the duration of postoperative hospital stay and each PFT value. The relationship between the parameters was analyzed using linear regression analysis.

PFT: Pulmonary Function Test; FESS: Functional Endoscopic Sinus Surgery
